# A case report of three people experiencing intractable autonomic dysreflexia following instillation of Uro-Tainer^®^ Polyhexanide 0.02%

**DOI:** 10.1038/s41394-024-00626-5

**Published:** 2024-04-05

**Authors:** Louise C. Kelly, Joanne V. Glinsky, Lisa A. Harvey

**Affiliations:** 1 John Walsh Centre for Rehabilitation Research, Faculty of Medicine & Health, University of Sydney, Sydney, NSW Australia; 2 Northern Sydney Local Health District, Sydney, NSW Australia

**Keywords:** Risk factors, Neurological manifestations

## Abstract

**Introduction:**

Historically, bladder washouts were used to instil therapeutic reagents directly into the bladder. This practice has expanded to include instillation of solutions that deal with catheter issues such as encrustation or formation of bio-film. They appear to provide a promising strategy for people with long term catheters. These products are readily available to purchase, but there is concern that people are using these solutions without a complete understanding of the purpose for the rinse and without clinical guidance to monitor response to treatment.

**Case presentation:**

These case studies include three people living with spinal cord injury (SCI) who developed severe autonomic dysreflexia (AD) when a catheter rinse was carried out using a particular solution. Each of the cases developed immediate and, in some cases, intractable AD requiring further intervention to resolve symptoms.

**Discussion:**

Catheter-associated urinary tract infection is a significant cause of morbidity and mortality in people living with SCI. Long-term catheters provide a vector for opportunistic micro-organisms to form bio-film and create an environment that promotes formation of struvite calculi, thus increasing the risk of chronic catheter blockage and urinary tract infection. Whilst these solutions are used to reduce these risks, they also pose additional risks to people susceptible to AD. These cases highlight the need for judicious patient selection and clinical oversight and management of adverse events when using catheter rinse solutions in certain people living with SCI. This is supported by a decision-making algorithm and a response to AD algorithm.

This case report was prepared following the CARE Guidelines (supplementary file 1).

## Introduction

‘Bladder irrigation’ and ‘bladder washout’ are terms frequently used to describe administration of therapeutic reagents into the bladder via an indwelling or suprapubic catheter [1–3]. The commonly used reagents in the 1970’s–1980’s were intravesical *oxybutynin*, *capsaicin* and 0.02% chlorhexidine. They were often inserted into the bladder with a ‘catheter’ syringe. Over recent years there has been increasing concern that this technique can create considerable pressure and suction and can damage the bladder mucosa [1, 4]. There has also been concern about the possible toxicity of these reagents on the urothelium [4–7]. These concerns have in part led to the development of pre-packaged bags of various solutions in volumes of up to 100 mL that are connected to the catheter. The content of the bag flows into the catheter with gravity by lifting the bag above the patient’s bladder. The height of the bag and the fullness of the bladder detmine how quickly the solution is instilled; however, the procedure generally takes 10-20 seconds. The passive flow of the solution limits the pressure and amount of solution entering the bladder. These bags are called Uro-Tainer(s)^®^ (B.Braun, Sempach, Switzerland) [8]. Uro-Tainer^®^ bags are approved for use as ‘catheter rinses’ around the world. Catheter rinses are for flushing the catheter. However, flushing an indwelling catheter invariably also involves adding quantities of solution to the bladder. For this reason, the distinction between a ‘catheter rinse’ and a ‘bladder irrigation’ or ‘bladder washout’ is ambiguous and the three terms are used interchangeably [4, 8–11].

**Fig. 1:**
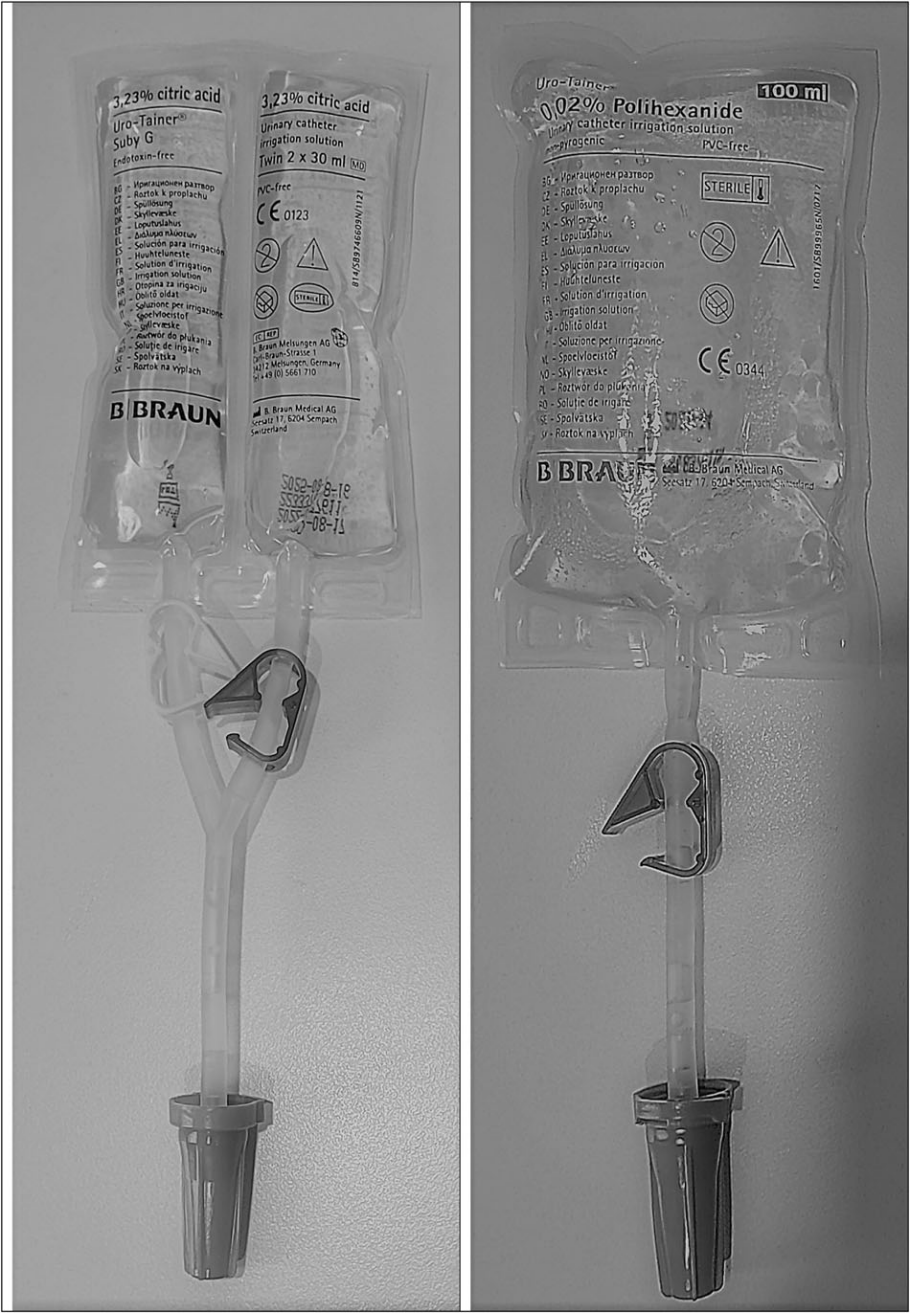
Uro-Tainer^®^ Suby-G and Uro-Tainer^®^ PHMB.

Recently in Australia, there has been a rapid increase in the use of two solutions with the Uro-Tainer^®^ bags in people living with spinal cord injury (SCI). They are (i) Twin citric acid 3.23% (Suby-G), and (ii) Polyhexanide 0.02% (PHMB) (Fig. 1). Suby-G is a an acidic solution designed to dissolve crystals and encrustations that may form in and around catheters [12]. Polyhexanide (PHMB) is a second-generation form of chlorhexidine which has been used as an antiseptic agent for the past 17 years. It is currently being used for the prevention of catheter-associated urinary tract infection (CAUTI) [13]. Unlike chlorhexidine, which was found to be ineffective against CAUTI and encouraged pathogen resistance [4, 14]; PHMB targets a broad range of bacteria as well as some fungal and protozoal organisms [13, 15].

Both Suby-G and PHMB in the Uro-Tainer^®^ bags are freely available for users to directly purchase from suppliers. However, there is little guidance about the appropriate use of these solutions. Consequently, there is a risk that these solutions are being misused without appropriate medical and/or nursing oversight, and they are being used for more than merely a ‘catheter rinse’ [16]. Furthermore, the instructions for use are not clear about the volume of solution that should be instilled, leaving the user with the impression that the entire 100 mL in the bag is to be inserted. This is potentially problematic because introducing too much volume into the bladder in a person living with SCI could cause bladder overdistention or overactivity, and mucosal irritation. In addition, these two products can cause autonomic dysreflexia (AD) (hyperreflexia) [12, 17].

Autonomic dysreflexia is an exaggerated sympathetic nervous system response to a noxious stimuli below the level of the individual’s neurological level. It is most commonly seen in people who have an SCI at or above the 6th thoracic nerve root injury, however, there are some cases where people below this level have experienced episodes (rarely past the 10th thoracic nerve root) [18]. The sequelae of uncontrolled AD can lead to catastrophic outcomes such as cerebral vascular accident or cardio vascular dysfunction as well as seizures [19]. No studies have investigated the possible link between catheter rinse solutions and AD although, Pannek et al, conducted a tolerability cohort study on Polyhexanide use in a select group of people living (81% had SCI or multiple sclerosis) with long term catheters. The authors reported 15 adverse events of bladder pain, irritation and spasm during instillation of polyhexanide but concluded that these were temporary. Of particular interest in the results is that the authors reported that 2 participants had a 40 mmHg and 70 mmHg rise in systolic blood pressure (SBP) (mean SBP 120.1 ± 17.5), but do not make any reference to a possible AD response [13].

In this paper, we present 3 cases of people who experienced intractable AD during and following the use of one of these solutions: PHMB. These cases highlight the need for judicious clinical assessment prior to recommending these products and careful nursing oversight when using them. The cases also highlight the need to clearly distinguish between ‘catheter rinses’ and ‘bladder irrigations’/ ‘bladder washouts’.

The demographic characteristics of the 3 case studies are outlined in Table 1. The initials used for each case are coded and do not represent any identifiable information of any of the cases. All blood pressure (BP) readings were taken with an automatic blood pressure monitor (model HEM-7121, Omron Health Care, Singapore) that provided a digital readout of BPs.

**Table 1:** Demographic information of cases.

Individual	Age	ISNCSCI	Time since injury (yrs)	Catheter rinse Solution	Indication for use	Prescribed by:
MK	59	C4 AIS A	41	PHMB	Mucous blocking SPC, CAUTI’s	Urologist
MS	44	C4 AIS A	5	PHMB	Blocking SPC, CAUTI’s	Continence Nurse Specialist
MB	47	T4 AIS B	9	PHMB	Blocking, SPC, CAUTI’s	Urologist

## Case presentations

### MK

MK was a 59-year-old female who sustained a C4 American Spinal Injuries Association Impairment Scale (AIS) A as defined by the International Standards for Neurological Classification of SCI (ISNCSCI) [20] SCI at the age of 18. She had a history of severe psoriasis, atrial fibrillation and pulmonary hypertension, and recurrent CAUTIs.

MK managed her bladder with a 18 Fg silver alloy coated suprapubic catheter (SPC) (Bardex I.C, BARD, Artarmon, NSW, Australia) on free drainage into a leg bag. She did not take any anticholinergic or β—3 adrenergic antagonist medication. She had experienced excessive sediment for several years and drank 4 L of water per day to control this. During a previous video urodynamic study, MK experienced severe AD after only 50 mL of contrast was instilled into the bladder. The procedure was immediately stopped, so it was not possible to get a diagnostic representation of her neurogenic bladder.

Multiple bladder calculi were identified during an admission to hospital for CAUTI in 2021, and MK was placed on a waitlist for surgery to have these removed via cystolitholapaxy. She was discharged home but then admitted again six weeks later for another CAUTI caused by extended spectrum beta lactamase (ESBL)/E-coli. Her admission to hospital was complicated by hyponatraemia. To treat the hyponatraemia, MK needed to reduce the amount of fluid that she consumed throughout the day. She was prescribed Suby-G to assist with managing the excessive sediment and mucous generated in her bladder. She was also prescribed intravesical instillations of *ropivacaine* 20 mL to manage her overactive neurogenic bladder instead of taking anticholinergic or β—3 adrenergic antagonist medication.

Upon discharge MK was referred to community nursing with a request for the nursing team to train her paid carer in the administration of Suby-G. There were no instructions about the quantity of solution to be used or the frequency of use. Therefore, the carer was instructed to administer Suby-G once per week, as per manufacturer recommendations.

The carer administered Suby-G following the manufacturer’s instructions [12]. Initially, the solution was warmed to body temperature to reduce the shock of cold fluid entering the bladder. The two bags of Suby-G were attached to the catheter and then raised (the bags and catheter are together referred to as a Uro-Tainer^®^). This allowed 30 mL of solution from the first bag to drain via gravity into the bladder. The solution was left for 5 min before the bag was lowered to drain the solution back into the bag. The process was repeated after clamping the first tube so as to use the 30 mL of solution from the second bag. An aseptic technique was maintained throughout. MK tolerated the Suby-G well. Therefore, Suby-G continued to be administered weekly without incidence for the next 4 months until a cystolitholapaxy was performed. Following the cystolitholapaxy, the Suby-G was ceased. However, 6 weeks after her cystolitholapaxy, MK contacted the community nurse. She reported that the sediment had reduced significantly following her surgery, but her catheter continued to block after 2 weeks due to mucous build up. She requested switching to PHMB to see if this would help. Polyhexanide (PHMB) is administered in a similar way to Suby-G but PHMB comes in a 100 mL bag and is drained immediately, rather than waiting for 5 min, as per the manufacturer’s instructions [17].

On the first attempt to use PHMB, MK experienced AD symptoms as soon as PHMB started to be inserted into the bladder (headache and flushing in the face). Her BP increased (190/130), and she required half a *glyceryl trinitrate* (GTN) tablet. In addition, a GTN patch was applied to MK’s upper chest after 6 min due to minimal response to sublingual treatment (174/122 mmHg). Her BP then trended back towards baseline, and the patch was removed at 20 min when MK’s BP was 100/84 mmHg.

Polyhexanide (PHMB) was tried again one week later. This time with careful monitoring. Her baseline BP prior to commencing was 129/82 mmHg. She felt comfortable to continue with the procedure, however, 2 min later, MK complained of a headache. Her BP was 180/137. A GTN patch was applied on her upper chest. After 10 min, MK’s BP was 188/135 mmHg therefore, 20 mL of *ropivacaine* was instilled into her bladder to provide topical local anaesthesia to the detrusor wall with the aim to reduce bladder irritation. After 20 min, her BP had returned to more acceptable parameters (117/96 mmHg) so the GTN patch was removed, however 10 min later, the patch had to be re-applied due to a rebounding BP of 201/137 mmHg. An additional half a GTN tablet was administered at 40 min as her BP was not settling. Ten minutes later, her BP was 170/85 mmHg, therefore a second instillation of *ropivacaine* was needed. After 5 min, MK’s BP was 112/69 mmHg. MK’s BP was monitored for a further 20 min and was stable with a maximum systolic BP of 114 mmHg. MK later reported that her BP was labile for 8 h following the procedure.

**Fig. 2:**
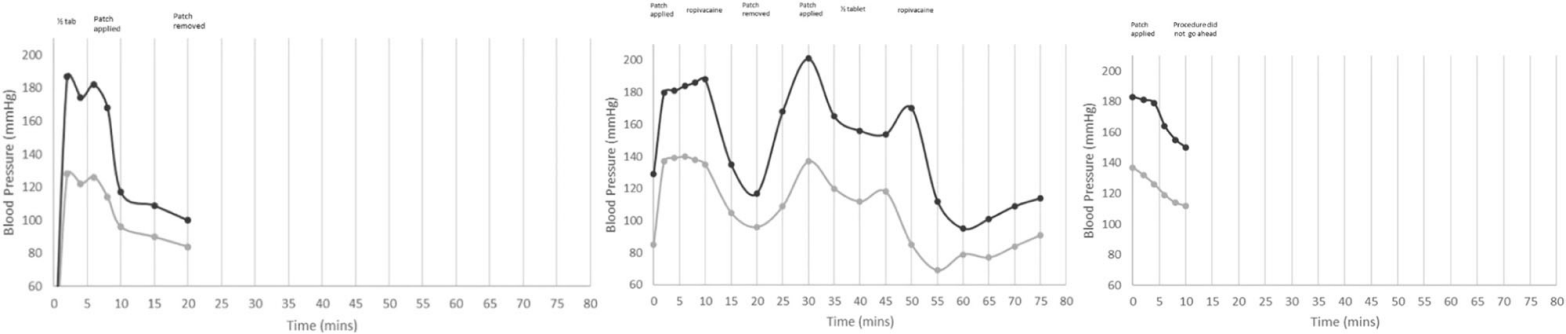
MK blood pressure response to three catheter rinse procedures.

There was one last attempt to administer PHMB one week later. However, MK’s BP prior to commencing the procedure was 183/137 mmHg (Fig. 2) and she described having irritation in her bladder, a headache and an irregular red rash started to appear on her face. A GTN patch was applied to her upper chest, however, her BP remained at 150/112 mmHg after 10 min so the rinse did not proceed. Catheter rinses with PHMB were ceased at this stage due to the risk of significant harm to MK secondary to the sequalae of intractable autonomic dysreflexia.

### MS

MS was a 44-year-old male who sustained a C4 AIS A SCI as per ISNCSCI [20] at the age of 39. He had a history of traumatic brain injury (TBI) (sustained at the time of the initial injury), mood disorder (effect of TBI), obesity, obstructive sleep apnoea, Type II Diabetes Mellitus and recurrent CAUTIs. MS reported having episodes of AD when experiencing CAUTI.

MS managed his bladder with a 20 FG open tipped silicone foley SPC (Supracath, Endotherapeutics, Macquarie Park, NSW, Australia) on free drainage into a leg bag. He took 5 mg *solifenacin* daily.

MS had a continence assessment completed by his Continence Nurse. MS told the nurse that he had experienced nine CAUTI’s and numerous SPC blockages in the past year. The nurse recommended that MS commence 50 mL PHMB three times per week to reduce the frequency of these blockages and infections.

MS used PHMB on three occasions. The procedure was performed by his regular community nurse. Each time, he developed pallor and piloerection of his arms and legs as well as flushing and sweating on his face and neck. He also complained of a sudden and increasingly severe headache. The details are as follows.

On the first occasion, MS’s systolic BP increased to 180 mmHg. His bladder did not drain any urine after 15 min and his AD symptoms did not resolve despite administration of GTN spray. At this point his catheter was changed (unplanned). His bladder started to drain immediately, and his BP returned to baseline within 20 min. The tip of the removed catheter was completely occluded with calcified sediment.

On the second occasion, MS again experienced AD symptoms and had a peak BP of 183/137 mmHg. However, this time, he responded to GTN therapy, and his AD resolved within 20 min.

**Fig. 3:**
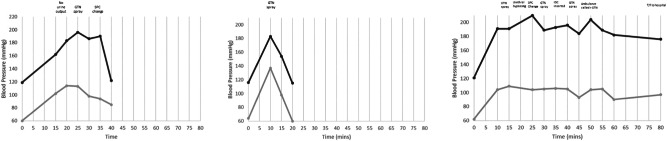
MS blood pressure response to three catheter rinse procedures.

On the third occasion, MS’s BP increased to 210/104 mmHg. He also experienced urethral bypassing in addition to AD symptoms. An unplanned SPC change was performed at this point, but it failed to drain, and his systolic BP remained above 190 mmHg. After 20 min an indwelling catheter (IDC) was also inserted via the urethra. These interventions did not resolve his AD and he had received the maximum recommended dose of GTN in the limited timeframe. An ambulance was therefore called, and MS was admitted for stabilisation of his AD symptoms (Fig. 3.). He was diagnosed with extreme bladder spasms causing mechanical obstruction of both his catheters. At this point, catheter rinsing using PHMB was ceased due to the significant risk of AD and MS was referred to a Urologist for investigation of his bladder overactivity.

### MB

MB was a 47-year-old male who sustained a T4 AIS B SCI as per ISNCSCI [20] at the age of 38. His injury was complicated by a severe brachial plexus on the right upper limb (dominant side) leaving him with contractures and a non-functioning right hand. MB had a history of type II Diabetes Mellitus, hypothyroidism, sleep apnoea, obesity (class II) and a stage IV pressure injury over his left ischial tuberosity. Additionally, MB had a previous history of renal and bladder calculi and recurrent CAUTI’s.

MB managed his bladder with a 24 FG open tipped, silicone foley SPC (Supracath, Endotherapeutics, Macquarie Park, NSW, Australia*)*. He did not take any anticholinergic or β – 3 adrenergic antagonist medication. The SPC was on free drainage into a leg bag that was emptied regularly. The leg bag was changed every 3 days (rather than weekly) as per MB’s preference. The SPC was changed every 4 weeks unless it became blocked, which was common during winter because MB reduced his fluid intake at this time. MB experienced AD when his SPC became blocked, however, he had some bladder sensation, so he was usually able to advise his carers to check the catheter drainage system for kinks or fullness.

MB’s Urologist recommended that he commence PHMB to manage the excessive debris and reduce bacterial loading and biofilm leading to CAUTI’s. He was instructed to use 50 mL PHMB three times per week.

On self-administration of PHMB, the nurse noted that MB winced with pain during the procedure and made many verbal sounds: indicative of pain. He then developed a very flushed and sweating face in addition to piloerection of his arms, torso and legs. His blood pressure was 176/104 mmHg. MB stated that his bladder was burning and stinging, he rated his pain as 8/10, but denied headache or his ‘usual’ AD symptoms. After 5 min, the pain had not subsided, and his BP was 196/115 mmHg. MB started to experience a headache and requested GTN, which was administered. After a further 5 min, MB was still experiencing 6/10 bladder pain. His BP was 185/114 mmHg. The nurse used 50 mL of sterile saline in a catheter tip syringe to rinse any residual solution from the bladder. An additional GTN was administered to help guard against an increase in AD symptoms. Five minutes later, MB reported a reduction in his pain (4/10) and his BP was 162/108 mmHg. His BP reduced further to 142/97 after 5 min and 123/76 after 10 min. His burning sensation also reduced to 2/10. MB was advised to take analgesia (*paracetamol or ibuprofen*) and increase his fluids to flush his bladder. The nurse arranged to provide carer education and training and a subsequent appointment was made.

On the second occasion, the nurse administered the PHMB to train MB and his carers. The timing of the procedure was scheduled to coincide with when MB changed his leg bag (once every three days) to reduce the risk of contamination.

**Fig. 4:**
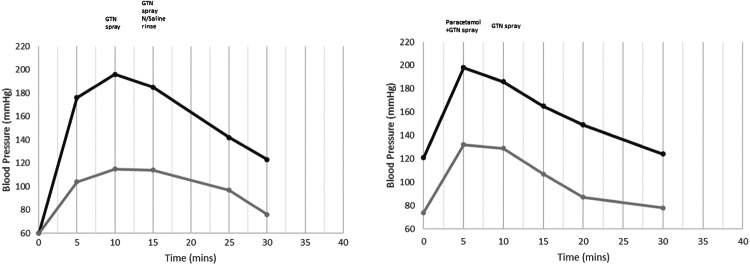
MB blood pressure response to two catheter rinse procedures.

Due to MB’s previous AD episode, his BP was taken prior to commencing the procedure. It was 121/74 mmHg. As the nurse administered the PHMB, MB felt immediate discomfort with the same type of pain as before (8/10). His BP was 198/132 mmHg and he demonstrated signs and symptoms of AD (Fig. 4). 1 g *paracetamol* and a spray of GTN were administered and MB was given fluids to drink. After 5 min his BP reduced to 186/129 mmHg. A further spray of GTN was administered. After 5 min MB reported that the pain had eased (6/10). His BP was 165/107 mmHg. The nurse suggested that the catheter be flushed with sterile saline via a catheter tip syringe, but MB declined because his AD was resolving. His BP reading was 149/87 mmHg (Fig. 4.) and following a further 10 min MB’s BP had returned to baseline readings of 124/78 mmHg. Further treatment with PHMB was discontinued because of the risk of AD.

## Discussion

This paper has identified the risks of using Uro-Tainer^®^ bags of PHMB as ‘catheter rinses’ in certain people living with SCI. In these three cases, a clinician with expertise in neurogenic incontinence recommended that the people commence using Suby-G or PHMB. However, the people were sent home to commence these solutions without any planned follow up. They were not always provided with any specific instructions about the quantity of solutions to use with the assumption that they are following the manufacturer’s instructions. This level of accessibility might portray a sense that these solutions and procedure for administering them are completely benign. Yet the manufacturers of Suby-G and PHMB warn that these solutions can cause irritation to the bladder and remind users to take precautions when using them in people at risk for AD [12, 17]. To ignore this risk could have catastrophic consequences for users and as these cases demonstrate, some clinical oversight is warranted, particularly with the PHMB solution that precipitated AD in all three cases.

Catheter associated urinary tract infections are a significant problem for people living with SCI who use permanent catheters to manage neurogenic bladder [9, 21, 22]. Genitourinary infection is the leading cause of hospital readmissions in this population across the world [23–33]. Infections related to catheters are associated with high levels of morbidity and mortality primarily since bacteria cannot be fully eradicated when a catheter is present in the bladder. This is due to micro-organisms adhering to the external and internal catheter surfaces, creating a protective biofilm for these organisms to proliferate and wait for an opportune time to infect the bladder [28, 34–40].

There is emerging evidence that use of ‘catheter rinse’ solutions such as Suby-G and PHMB may have benefit in reducing CAUTI and preventing catheters from blocking [10, 39, 41–43]. However, a Cochrane review by Shepherd et al., concluded that of the 7 studies that met the inclusion criteria, they found methodological flaws in design and small numbers. Ultimately they concluded that there is insufficient evidence to support the efficacy of these solutions [3].

Currently, there are no clinical trials demonstrating the therapeutic effects of Suby-G and PHMB. Instead, their use has been based upon in-vitro or animal studies [2, 6, 11, 14, 39, 43–46]. Andersen et al., have recommended more high-quality trials investigating the effectiveness of PHMB [39]. There is a current study comparing citric acid to normal saline to no ‘catheter rinse’ in a range of people including people with SCI. [9]. The researchers are using 100 mL of 0.9% Normal Saline or x2 30 mL 3.23% citric acid with a third arm which is the control group. The results of this study will be important for clarifying whether citric acid has any positive effects on reducing blockages and symptomatic CAUTI. Interestingly, this study explicitly excludes people at risk of AD (anyone with a lesion at or above T6). Future studies will need to clarify precautions around the use of these solutions in people living with SCI and specifically people at risk of AD. Future studies should also include bladder spasm, irritation, pain and AD as outcome measures as these solutions are known to have the potential to cause irritation to the bladder wall [4, 7, 12, 17, 42].

A limitation of this case series is that the adverse reaction of intractable AD was only observed in these three people living with SCI and only with PHMB usage. MK was reported to react adversely to both 50 mL of contrast and 50 mL of PHMB, so the volume of solution could have been the causative factor for developing AD, rather than the active ingredient in the solution. So, we are not inferring that these solutions are injurious especially as we cannot exclude underlying bladder pathology affecting bladder capacity, tendency to spasm or urothelial irritation in the absence of evidence of symptomatic UTI, as in MK’s case. Instead, we are recommending that if catheter rinse solutions are going to be used in people living with SCI, consumers and community clinicians would benefit from clear guidance on the indications for use, frequency of use and how to use the products. People at risk of AD would need a neurogenic bladder assessment, RN supervision to monitor BP and an AD plan in place prior to commencing. It is important to establish clear guidance on the amount of solution that is appropriate for ‘catheter rinses’. An example of such guidance is provided (supplementary file 2).

## Conclusion

Whilst catheter rinse systems may have a role in reducing CAUTI or catheter blockages associated with long term catheter use, there needs to be more clinical oversight when considering use of these solutions, and more careful monitoring when using these solutions, especially in people living with SCI and at risk of AD.

### Supplementary information

Supplementary File 1.

Supplemantary File 2.

## Data Availability

All available individual data are provided in the paper.
