# Supplementary material for: A case report of three people experiencing intractable autonomic dysreflexia following instillation of Uro-Tainer® Polyhexanide 0.02%
Source: Spinal Cord Ser Cases. 2024 Apr 5;10:17. doi: 10.1038/s41394-024-00626-5 (PMC10997763; doi:10.1038/s41394-024-00626-5)
Supplement: Supplementary file 1 [file 41394_2024_626_MOESM1_ESM.pdf]

Supplementary File 1. Equator Network CARE Case Report Guidelines Checklist

Topic	Item No.	Description	Reported on Line Number/s
**Title**	1	The diagnosis or intervention of primary focus followed by the words “case report”	Title Page
**Keywords**	2	2 to 5 key words that identify diagnoses or interventions in this case report, including "case report"	Title page
**Abstract**	**3a**	Introduction: What is unique about this case and what does it add to the scientific literature?	1-7
	**3b**	Main symptoms and/or important clinical findings	8-11
	**3c**	The main diagnoses, therapeutic interventions, and outcomes	8-11
	**3d**	Conclusion—What is the main “take-away” lesson(s) from this case?	12-20
**Introduction**	4	One or two paragraphs summarizing why this case is unique?	48-74
**Patient Information**	**5a**	De-identified patient specific information	75-76
	**5b**	Primary concerns and symptoms of the patient	117-140, 153-172, 183-207
	**5c**	Medical, family, and psycho-social history including relevant genetic information	81-88,141-145, 173-183
	**5d**	Relevant past interventions with outcomes	88-102,104/5,
**Clinical Findings**	6	Describe significant physical examination (PE) and important clinical findings	139-140, 145, 175-179
**Timeline**	7	Historical and current information from this episode of care organized as a timeline	80-215
**Diagnostic Assessment**	**8a**	Diagnostic testing (such as PE, laboratory testing, imaging, surveys	N/A
	**8b**	Diagnostic challenges (such as access to testing, financial, or cultural)	N/A
	**8c**	Diagnosis (including other diagnoses considered)	N/A
	**8d**	Prognosis (such as staging in oncology) where applicable	N/A
**Therapeutic Intervention**	**9a**	Types of therapeutic intervention (such as pharmacologic, surgical, preventive, self-care)	101,110,117,123,135, 157, 162, 164, 191. 204
	**9b**	Administration of therapeutic intervention (such as dosage, strength, duration)	112, 114, 118/9,122/3, 157-170,189
	**9c**	Changes in therapeutic intervention (with rationale)	N/A
**Follow up and Outcomes**	**10a**	Clinician and patient-assessed outcomes (if available)	128-133, 167-170, 215
	**10b**	Important follow-up diagnostic and other test results	N/A
	**10c**	Intervention adherence and tolerability (How was this assessed?)	80-215
	**10d**	Adverse and unanticipated events	80-215
**Discussion**	**11a**	A scientific discussion of the strengths AND limitations associated with this case report	235 - 263
	**11b**	Discussion of the relevant medical literature with references	216-263
	**11c**	The scientific rationale for any conclusions (including assessment of possible causes)	258-263
	**11d**	The primary “take-away” lessons of this case report (without references) in a one paragraph conclusion	265 – 268
**Patient Perspective**	12	The patient should share their perspective in one to two paragraphs on the treatment(s) they received	112-114, 125, 133, 136, 191-203, 208-213
**Informed Consent**	13	Did the patient give informed consent? Please provide if requested	YES
