# Supplementary material for: A case report of three people experiencing intractable autonomic dysreflexia following instillation of Uro-Tainer® Polyhexanide 0.02%
Source: Spinal Cord Ser Cases. 2024 Apr 5;10:17. doi: 10.1038/s41394-024-00626-5 (PMC10997763; doi:10.1038/s41394-024-00626-5)
Supplement: Supplementary file 2 — Supplemantary File 2. [file 41394_2024_626_MOESM2_ESM.pdf]

Decision making tool when considering commencement of Urotainer® in people with SCI.

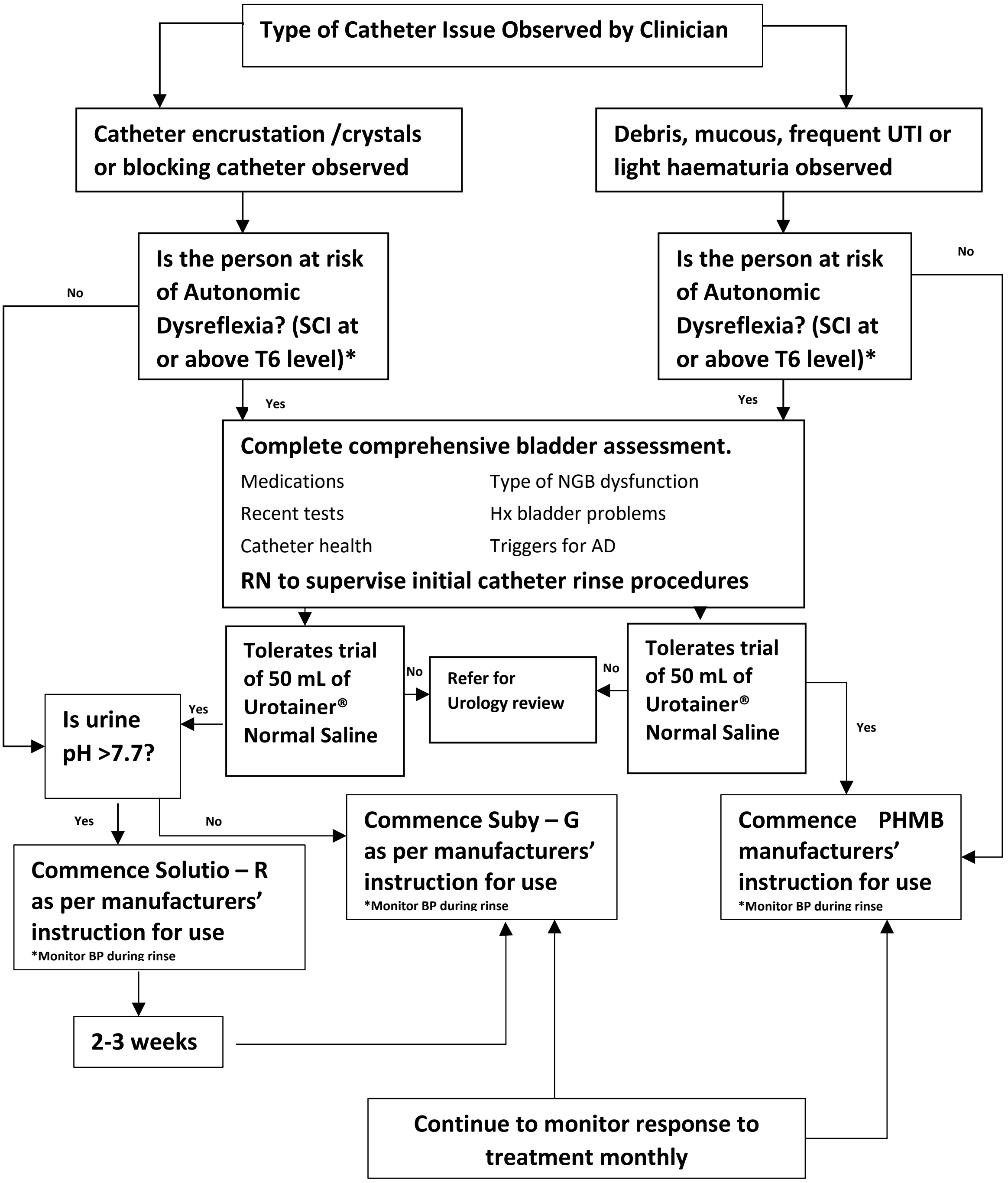

Recommended Volumes for Rinse:			
**Solution Type**	**Packaging**	**Recommended vol mL**	**Time in bladder**
**Suby-G Twin**	X2 30 mL	X2 30 mL	5 min
**Solutio-R Twin**	X2 30 mL	X2 30 mL	5 min
**Suby-G**	100 mL	50 mL	5 min
**Solutio-R**	100 mL	50 mL	5 min
**PHMB**	100 mL	50 mL	in/out

Algorithm developed with reference to: BBraun. Uro-tainer® catheter maintenance solutions. A guide to the right management plan. Norwest, NSW: BBraun Australia, 2020.
